# Chaotic expression dynamics implies pluripotency: when theory and experiment meet

**DOI:** 10.1186/1745-6150-4-17

**Published:** 2009-05-15

**Authors:** Chikara Furusawa, Kunihiko Kaneko

**Affiliations:** 1 Department of Bioinformatic Engineering, Graduate School of Information Science and Technology, Osaka University, 1-5 Yamadaoka, Suita, Osaka 565-0871, Japan; 2 Department of Basic Science, University of Tokyo, Komaba, Meguro, Tokyo 153-8902, Japan; 3 Complex Systems Biology Project, ERATO, JST, Komaba, Meguro, Tokyo 153-8902, Japan

## Abstract

**Background:**

During normal development, cells undergo a unidirectional course of differentiation that progressively decreases the number of cell types they can potentially become. Pluripotent stem cells can differentiate into several types of cells, but terminally differentiated cells cannot differentiate any further. A fundamental problem in stem cell biology is the characterization of the difference in cellular states, e.g., gene expression profiles, between pluripotent stem cells and terminally differentiated cells.

**Presentation of the hypothesis:**

To address the problem, we developed a dynamical systems model of cells with intracellular protein expression dynamics and interactions with each other. According to extensive simulations, cells with irregular (chaotic) oscillations in gene expression dynamics have the potential to differentiate into other cell types. During development, such complex oscillations are lost successively, leading to a loss of pluripotency. These simulation results, together with recent single-cell-level measurements in stem cells, led us to the following hypothesis regarding pluripotency: Chaotic oscillation in the expression of some genes leads to cell pluripotency and affords cellular state heterogeneity, which is supported by itinerancy over quasi-stable states. Differentiation stabilizes these states, leading to a loss of pluripotency.

**Testing the hypothesis:**

To test the hypothesis, it is crucial to measure the time course of gene expression levels at the single-cell level by fluorescence microscopy and fluorescence-activated cell sorting (FACS) analysis. By analyzing the time series of single-cell-level expression data, one can distinguish whether the variation in protein expression level over time is due only to stochasticity in expression dynamics or originates from the chaotic dynamics inherent to cells, as our hypothesis predicts. By further analyzing the expression in differentiated cell types, one can examine whether the loss of pluripotency is accompanied by a loss of oscillation.

**Implications of the hypothesis:**

Recovery of pluripotency from determined cells is a long-standing aspiration, from both scientific and clinical perspectives. Our hypothesis suggests a feasible route to recover the potential to differentiate, i.e., by increasing the variety of expressed genes to restore chaotic expression dynamics, as is consistent with the recent generation of induced pluripotent stem (iPS) cells.

**Reviewers:**

This article was reviewed by David Krakauer, Jeroen van Zon (nominated by Rob de Boer), and Williams S. Hlavacek.

## Introduction and background

During normal development, cells undergo a unidirectional course of differentiation that progressively decreases the number of cell types they can potentially become. Totipotent cells in early embryos can differentiate into any of the cell types that make up the adult organism, but lineage-specific multipotent stem cells have the potential to produce only a limited number of cell types. Further development generates determined cells that cannot differentiate any further.

**Figure 1:**
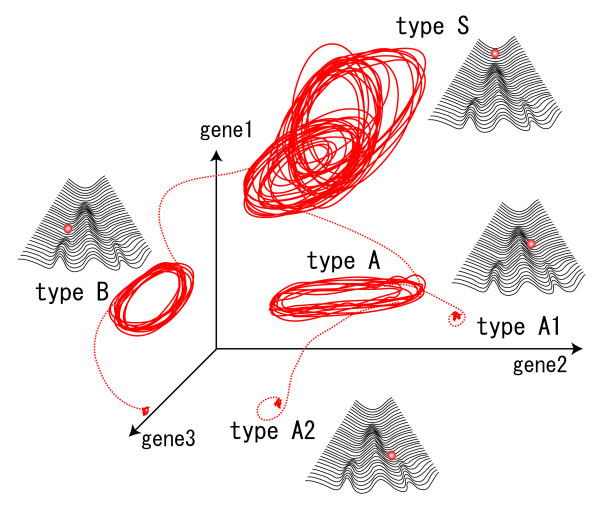
**A Schematic representation of phase-space which represents expression dynamics of several cell types**. Each axis shows the expression level of a gene. The trajectory in the phase space represents the time course of expression profile, where attractors correspond to distinct cell types, i.e., type S, A1, A2, and B. In this example, type-S cells act as stem-type cells, which can differentiate into type-A, B, while differentiated types lose the potential to differentiate into other cell types. The epigenetic landscape of cellular states is also shown, in which the change of cellular state is represented as the trajectory of a ball falling along branching valleys.

The degree of multipotency is in general established by intracellular states, including gene expression patterns, protein concentrations, epigenetic factors, and so forth. Thus, is it possible to characterize pluripotency in terms of such factors? How is the loss of multipotency driven by changes in the cellular state? A long-standing theoretical basis for the process of successive determination from pluripotent states is Waddington's epigenetic landscape [1], in which the cell differentiation process is represented as the trajectory of a ball falling along branching valleys (see Figure 1). Although this description has been influential for decades, it remains rather qualitative, and it is important to establish a more quantitative connection to cellular state dynamics.

A recently developed dynamical systems model of cells attempts to quantitatively answer the raised questions by accounting for the loss of pluripotency resulting from cell differentiation [2-4]. By carrying out simulations with thousands of gene expression networks, we found that irregular oscillations, including chaotic dynamics (as will be described below), in gene expression patterns lead to pluripotency. However, recent progress in stem cell biology has revealed that the intracellular states of pluripotent or multipotent stem cells are heterogeneous [5-7]. For example, Hayashi et al. [5] recently observed that the expression level of Stella, which is a marker of pluripotency and germ cells, is heterogeneous within an embryonic stem cell population. Chang et al. [7] recently showed that gene expression levels slowly itinerate over several quasi-stable states for hematopoietic progenitor cells. On the basis of our simulation results and these experimental reports, we here propose the hypothesis that itinerant dynamics caused by "chaotic" oscillations in gene expression levels lead to pluripotency. As defined in dynamical systems theory, chaos is a dynamic state with irregular but ordered oscillation that allows for a variety of state changes, while it is an attractor and is stable against perturbation [8-10]. With this stability, a proliferation of cells with chaotic dynamics is possible, and with the stochastic behavior generated by its dynamics, probabilistic differentiation to other types of cells is possible. With this hypothesis, we also intend to establish a close correlation between experimental results and dynamical systems theory, to characterize pluripotency.

## Proposition of the hypothesis

Representing the cellular state involves a huge number of variables, including global gene expression, concentrations of various proteins and metabolites, and epigenetic factors such as DNA methylation. As depicted in Figure 1, the cellular state at a particular point in time is represented by a point in a multidimensional phase-space in which each axis represents a variable (*e.g*., the expression level). The temporal evolution of the cellular state generates trajectory in the phase-space. As a consequence of intracellular reaction dynamics and cell-cell interactions, the cellular state is directed into a particular restricted domain, referred to as an "attractor" in dynamical systems theory (as discussed by Kauffman and others [11,12]). The attractor can be a fixed point if the cellular state does not change over time, but it can be a closed cycle when the cellular state oscillates. Depending on the initial conditions, cells can be driven toward several attractors that produce different cell types. However, the cell differentiation process progresses through cell-cell interactions, and the change and selection of cellular states are a result of inter- and intracellular dynamics propelling the cellular state toward one of a number of attractors.

This "dynamical systems" viewpoint provokes two important questions. First, how can the variance between multipotent and differentiated cellular states be explained in terms of the different characteristics of attractors? Second, how can the irreversibility of cellular determination be described in terms of high-dimensional phase-space dynamics?

To address these questions, we examined a simple model of interacting cells that describes intracellular protein expression dynamics as influenced by a large number of randomly connected genes [2-4]. We conducted thousands of simulations of this class of models, using different rules for cell-cell interaction and a variety of intracellular gene networks and parameters. We found that "differentiation," i.e., transition between attractors, generally occurs by perturbation due to cell-cell interaction. When the number of cells increases as a result of cell division, cell-cell interactions become stronger and the intracellular states of some cells are destabilized. When this occurs, some cells are deflected away from the original attractor and differentiate into another state that has different gene expression patterns, as displayed in Figure 1. The emergence of new cell types stabilizes the dynamics of the other cell types. The path to each potential state is generated as a bifurcation leading toward a new attractor, as in the motion of a ball in Waddington's picture of gene expression.

As shown in the Figure, the initial cell type, depicted as "type S," exhibits irregular oscillation (i.e., chaos) in the expression levels of a certain gene. The cellular state wanders through different levels of gene expression in time, exhibiting a large degree of temporal variation. In spite of such variation, the expression level stays within some region, an attractor. When the number of type S cells increases through cell division, some cells differentiate into other cell types that have distinct gene expression profiles, depicted in the Figure as type A and type B. Following increases in their numbers, type A cells further differentiate into type A1 and type A2 cells, which progress and terminate in a state no longer capable of differentiating into other cell types. Here, the type S pool acts as a multipotent source that can generate all cell types in the system. The decision as to whether a stem cell proliferates or differentiates appears to be a stochastic process, arising from chaotic intracellular expression dynamics. However, the rate of differentiation or proliferation is regulated by cell-cell interactions, as this stochasticity is generated by chaotic intracellular dynamics.

The initial attractor S has variation in many directions in the state space, and involves a larger number of expressed genes, whereas differentiated cells A or B have biased expressions represented by lower-dimensional attractors. Types A1 and A2 have further biased expressions. As the developmental process progresses, the diversity in chemical compositions is lost successively. This theoretical conclusion is consistent with the experimental observation that in multipotent cells, relatively large numbers of genes are weakly expressed, whereas differentiated cells express a smaller number of lineage-specific genes strongly, (e.g., [13]). With this loss of diversity, the reaction dynamics become simpler, so that the oscillatory dynamics of the original cell types is weakened successively with differentiation, until it is lost in the terminally differentiated cell type. Hence irreversible loss of potency is characterized by a reduction in both the diversity and fluctuation of gene expression. This theoretical argument was confirmed in our numerical experiments.

On the basis of these theoretical results, we propose the following "chaotic itinerancy" hypothesis for pluripotency.

*The expression levels of some genes exhibit irregular oscillation in pluripotency, known as chaotic dynamics. As cells lose pluripotency, the gene expression dynamics loses "chaoticity," and get simpler, until the oscillation is lost in committed cells. With chaotic dynamics, gene expression levels of pluripotency itinerate over several quasi-stable states. As development progresses, each of these quasi-stable states is modified and stabilized, leading to differentiated cell types. This chaotic dynamics introduces variety in state changes and leads to probabilistic differentiation from stem cells. The chaotic dynamics of pluripotent cells involves changes of expression levels of several genes, whose number (effective degrees of freedom in the sense of dynamical systems) decreases as differentiation progresses*.

## Testing the hypothesis

As mentioned above, heterogeneity in gene expression levels is experimentally observed in homogeneous stem cell populations. According to our predictions, high-dimensional irregular oscillatory dynamics in gene expression is the origin of such heterogeneity, instead of stochastic noise known to be associated with the gene expression machinery [14]. Because gene regulatory dynamics is generally interconnected, we expect that such irregular oscillation should be widely observed in expression levels of many genes in stem cells, and especially for those closely related to differentiation from stem cells, such as Oct3/4 and Sox2 in the case of embryonic stem cells. To test the hypothesis, it is crucial to measure the time course of gene expression levels at the single-cell level. By analyzing time series of single-cell-level expression data, one can extract information about the attractor of the corresponding cell type. For example, one can distinguish whether the variation of expression level over time is due only to stochasticity in expression dynamics, or originates in the chaotic dynamics inherent to cells, by using standard time-series analysis such as the power spectrum, embedding dimension, and Lyapunov exponents [15].

Fluorescence-activated cell sorting (FACS) analysis should also be a powerful method to test our hypothesis. For example, using FACS analysis Chang et al. [7] reported slow itinerant dynamics of gene expression patterns over quasi-stable states, which suggested the existence of high-dimensional slow dynamics in the gene regulatory network. To further the study, the use of two- or three-color FACS analysis of genes that are related to pluripotency will be important because such data will provide information about the "trajectory" in multidimensional expression dynamics. For instance, by sorting cells having rather homogeneous intracellular states from a population of pluripotent cells and later following the development of single cells, one can trace the trajectory of cellular states in the multidimensional phase-space, as depicted in the Figure. Our hypothesis predicts the existence of complex expression dynamics in stem cells and reduced complexity in differentiated cells.

To further test if chaotic dynamics in stem cell systems can exist generally in gene expression dynamics and if it can be achieved through evolution, we have recently investigated what type of cellular dynamics are adopted to allow for differentiation to multiple cell types. By taking a huge number of different gene regulation networks, we have made computer simulations of interacting cells with on/off switching expression dynamics governed by the network. For most of the networks cell differentiation is not observed, but for a few of them, differentiation to multiple cell types progresses with the developmental process to increase the cell number. The latter gene regulatory networks which can generate multiple cell types are selected by using a genetic algorithm (GA) with cell type heterogeneity as the fitness function, and adding mutation to the network at each generation. As results, we found that most of the selected regulatory networks show complex oscillatory dynamics as chaotic dynamics in the early stage of development, from which multiple cell types with fixed gene expression patterns are differentiated. Hence, stem-type cells with chaotic dynamics are ubiquitous. Even though chaotic expression dynamics are rather rare in randomly connected regulatory networks, most of the networks evolved under the fitness condition adopted chaotic dynamics to achieve cell type heterogeneity. Another possible mechanism for cell differentiation that adopts simple bistable or multistable dynamics was very rarely observed, probably because it needs fine tuning of control parameters as will be discussed later.

## Implications of the hypothesis

The source of the heterogeneity of cellular state in pluripotent stem cells still remains unclear [5-7]. One possible source is the noise in expression dynamics due to fluctuations in mRNA and protein numbers [14], which, however, might have difficulty explaining why the heterogeneity is larger in stem cells than in committed cells. Here we propose another source of cellular heterogeneity: chaotic dynamics in intracellular dynamics. One significant advantage of this chaos hypothesis is that it naturally leads to regulation of the rate of differentiation from the stem cells. If the heterogeneity were solely due to noise, it would be difficult to regulate the rate.

For example, to explain differentiation processes, one could adopt a simple bistable or multistable dynamics in which transitions among states are driven by noise. However, to control the probability of differentiation in such a system, the amplitude of the noise needs to be finely tuned, depending on the external environment such as the states of surrounding cells. Robust differentiation into more than two cell types would be difficult, since to maintain a population ratio of all the cell types, multiple parameter values to control the magnitudes of the noise and gene expression dynamics have to be finely tuned. Such control of several parameters, in particular noise amplitudes, would be quite difficult.

In contrast, the chaotic dynamics depends on the intracellular state, which leads to the regulation of the differentiation ratio, and then to the robustness of the cell type population ratios. As shown in Ref. [3], when some type of differentiated cell is removed from a cell population, the trajectory of chaotic expression dynamics in stem cells is modulated according to the resulting change in cell-cell interaction. This modulation then leads to an increase in differentiation rate from the stem to the removed cell type, so that the original population ratio is recovered. This regulation of differentiation rate to maintain the robustness of cell populations is a natural consequence of the interaction-based differentiation mechanism we propose. In contrast, if the cellular heterogeneity in stem cells were solely due to the noise in expression dynamics, the control of differentiation rate would be difficult, and the developmental process would not be robust. Such regulation of differentiation rate would be important for robustness in the development and maintenance of multicellular systems. Confirmation of such regulation by measuring the differentiation rates under controlled number ratios of each cell type should be important to test this implication [16].

Recovery of pluripotency from determined cells is a long-standing aspiration, from both scientific and clinical perspectives. In plant cells, recovery of pluripotency has been relatively easy to achieve, but in animal cells, nuclear transplantation techniques were needed to reverse the loss of pluripotency [17]. As long as cells are considered as dynamical systems consisting of many components such as genes and proteins, restoration of pluri- or multipotency should be possible, *in principle*, by suitably changing the composition of these factors, i.e., the location of the trajectory in the multidimensional space of the theory. However, a practical solution to recovery of pluripotency will require further understanding of intracellular dynamics.

Our hypothesis suggests a feasible way to recover pluripotency. By recovering larger degrees of freedom in intracellular dynamics via activating the expression of multiple genes, multipotency can be restored. This theoretical prediction is consistent with recent observations of the generation of induced pluripotent stem (iPS) cells from fibroblasts by expressing a small number of critical regulatory genes [18]. According to our hypothesis, it will be important to examine recovery of temporal oscillation as well as the diversity in gene expression levels after recovering multipotency by generation of iPS cells. For the theory, the goal should be to determine the optimal number of activated genes required to reverse the arrow of time in terms of pluripotency.

## Competing interests

The authors declare that they have no competing interests.

## Authors' contributions

CF and KK conceived and designed the project. CF and KK wrote the paper and approved the final manuscript.

## Reviewer's comments

### Reviewer's report 1

Dr. David Krakauer (Santa Fe Institute, Santa Fe, New Mexico, USA)

#### Reviewer Comments

This is a short hypothesis paper summarizing previous published work. No technical details are provided in this paper, but as far as I can tell, the central idea has been previously published (available on the arXiv) as the "chaos hypothesis" of irreversible differentiation and is represented by two papers cited in the bibliography. I am not able to see what is new in this submission other than removing some of the math, and describing a few experimental expectations of the model.

##### Author's response

When we proposed the original chaos hypothesis about a decade ago [2,3], the time-lapse measurement of gene expression in a cell was rather limited. For instance, time-series analysis of gene expression at a single cell level was not available. At that time, few took existence of oscillatory gene expression seriously, but as a result of drastic progress in experimental technique within a decade, the existence of oscillatory expression dynamics itself is no longer doubted (e.g., [19]). Now the time is ripe to compare our hypothesis with experimental results quantitatively. Hence, we have updated the original hypothesis by adding the following three points. First, we present a novel interpretation of recent experiments on the basis of our theory. As mentioned in the manuscript, the heterogeneity of gene expressions in stem cells has attracted attention (Refs. [5-7]), and thus discussing the origin of this heterogeneity in the light of the chaos hypothesis is meaningful. Second, on the basis of the advances in gene expression measurements, we propose possible ways to confirm our hypothesis. For example, the time-series analysis of gene expression at the single cell level can provide information about the characteristics of intracellular dynamics. Do the gene expression dynamics of a stem cell show irregular oscillation as expected by chaos? Does such oscillation disappear in committed cells? We address such questions here and propose experiments to answer these questions. Third, to compare with these expression data, we have also carried out extensive numerical simulations of a novel model that explicitly takes into account biological gene expression dynamics rather than the original abstract models adopted a decade ago. The results of these novel simulations demonstrate the universality of chaotic gene expression dynamics for cells that show differentiation, and then enable us to explicitly relate gene-regulatory dynamics in the model to experimental observations. The general relevance of our chaos hypothesis is confirmed by these simulations. In the revised manuscript, we have added some remarks on the recent results briefly in the last paragraph of the "Testing the hypothesis" section. The above three points provide a new insight into pluri- and multipotency in stem cell systems, and thus are worth publication as a hypothesis article.

#### Reviewer Comments

The authors seek to explain the loss of pluripotency during development. The idea is that undifferentiated cell states represent an itinerant, chaotic regime in gene expression dynamics. During development, feedback from growing populations of cells, reduces the effective dimensions of gene expression, thereby fostering terminal differentiation. This is an interesting idea. The hypothesis requires that variation in gene expression should be greater for pluripotent cells, and manifest as a slow transit among alternative activation states. Later generations of cells should show a more limited expression repertoire. One concern I have is that the authors do not seem to be very familiar with the growing literature on stem cells. Much of this literature questions the whole idea of terminal differentiation. One of the pioneers in this field, Irving Weissman has over the course of many years, demonstrated that the cellular context is as important as internal gene expression arising from division stage in generating specificity. Thus the loss of plasticity is often only a confound of context and not a property of the cell. I would request that the authors read at least a few of the review papers from the Weissman lab. e.g. *Cell*, *100:157*-*168 *(*2000*)

##### Author's response

We are familiar with recent experiments. Indeed, the recognized importance of the cellular context in developmental processes provides additional evidence in support of our hypothesis. In our model simulation, some cell types can appear only when these cells interact with other cell types. For example, the state of type-B cells can be maintained by interaction with type-S and type-A cells, and when a type-B cell is isolated from other cells it goes to another cell type. Thus, to achieve a cell society consisting of type-S, A, and B cells, the order of emergence of these cell types is important (in this case, type-S cells appear first, and after increase of type-S cells by cell divisions, they differentiate into type-A and B cells simultaneously). Another example of such cellular context dependency in our simulation can be seen in the "community effect." When we transplant a single differentiated cell to another cell society, the transplanted cell changes its cellular state in accordance with the states of surrounding cells. In contrast, when a group of differentiated cells are transplanted, they can keep their original state. Such a community effect means that the behavior of a cell in our model simulation deeply depends on the context of surrounding cells. Note that such a community effect is widely seen in developmental processes (e.g., [20]).

Our dynamical systems model provides a novel description of cellular context in the developmental process. In our scheme, a cellular state is represented by an attractor in a high-dimensional dynamical system. However, an attractor corresponding to each cell type is not invariant against possible changes of surrounding cells, and instead is modified by the cell-cell interaction. This modification leads to context dependence in a cell society as mentioned above. According to our theory, cellular states are sustained through cell-cell interactions with each other, which results in the cellular context dependency and the robustness of a cell society.

#### Reviewer Comments

I found that test of the hypothesis a little cursory. The paper needs to show the results of some form of reconstruction of effective dimensionality for a system that is known to be itinerant and stochastic. In other words, given that many simulations have already been performed, as described by the authors, please show us how the stated hypothesis differs from a family of alternative null models. I think this is a minimal requirement for this paper to be compelling. In particular, can the hypothesis be tested and validated in the face of both intrinsic and extrinsic noise?

##### Author's response

We have already discussed how noise in intracellular dynamics affects the proposed differentiation process in our previous paper [21,3]. We have performed the simulations with noise in the dynamics and found that the interaction-based differentiation process is robust to noise of a certain amplitude. On the basis of the simulations, we roughly estimated that the minimal number of proteins necessary for robust development is around 100–1000, which might be consistent with observations in experiments. Chaos in expression dynamics has two faces, that is, stability as an attractor and instability leading to stochasticity. Robustness to intrinsic and extrinsic noise is a natural consequence of the first aspect.

Now we compare our dynamic differentiation model with a possible alternative model that might be adopted for cell differentiation. A typical model for differentiation adopts bistable dynamics in which transitions between states are driven by noise (e.g., [22]). Here we discuss the merits of our mechanism in comparison with such a bistability mechanism.

First, for switching mechanisms driven by noise, it is difficult to explain the robustness of cell type populations required for robust development. Of course, a stable number distribution could exist if the switching between the states is frequent enough to maintain the equilibrium distribution. This would mean that each cell state (even of a committed cell type) would be metastable and transformed often. This contradicts experimental observations of stable committed cell types in real stem cell systems. In our model, in contrast, the probability of a stem cell's differentiation changes depending on "cellular context." Such regulation of differentiation ratio according to cell-cell interaction is a natural outcome of our theory. However, to control the probability of differentiation in a bistable system, the amplitude of the noise needs to be finely tuned, depending on the external environment, such as the state of surrounding cells. Such control of noise amplitude would be quite difficult.

Second, robust differentiation into more than two cell types by combination of the bistability mechanisms would be even more difficult. Consider a case of differentiation into three cell types. To maintain a population ratio of cell types, at least three parameter values to control the magnitudes of the noise have to be elaborately controlled in addition to several other parameters to control gene expression dynamics. Hence, a finely tuned and sensitive regulation mechanism would be required to have a stem-cell system with differentiation into several cell types. In contrast, we proposed that interacting cells with chaotic expression dynamics can show differentiation into multiple cell types without tuning parameters. According to our theory, the robustness of cell population ratio generally appears without any sophisticated control. This spontaneous regulation of differentiation frequency is one advantage of our hypothesis over a mechanism with stochastic switching among multistable fixed states.

The third merit we have recently confirmed by numerical simulations concerns the evolvability of stem cell systems. We have recently investigated what type of cellular dynamics is most likely to generate multiple cell types, by using computer simulations of interacting cells with on/off switching expression dynamics. In the simulations, the gene regulatory networks that can generate multiple cell types are selected by using a genetic algorithm (GA) by imposing the fitness function of increasing cell type heterogeneity. Through extensive simulations, we have confirmed that the selected regulatory networks generally show chaotic dynamics in their stem-type cells. Most of the networks evolved under the fitness condition adopted chaotic dynamics to achieve cell type heterogeneity. Instead of bistable mechanisms that need fined tuning of parameters, our chaos mechanism was selected through evolution.

To clarify the advantage of the proposed hypothesis relative to a simple bistable switch, we added the above discussion briefly into the first and second paragraphs of the "Implications of the hypothesis" section.

#### Reviewer Comments

One confusion that I have is why/how stem cells maintain a coherent phenotype when their gene expression is chaotic?

##### Author's response

This is simply due to the general nature of deterministic chaos. Chaos in dynamical systems is an "attractor" and is a stable state. Even after such a state is perturbed externally, it can come back to the original attractor. The trajectory of a chaotic cellular state occupies a finite portion of the phase space as shown in Figure. 1, to which nearby trajectorys are attracted. Thus, when we measure such chaotic dynamics, a coherent phenotype should be found, even though there remains phenotype variation due to the chaotic dynamics at each instant. Indeed this variation explains well the cellular heterogeneity experimentally observed in stem cells.

#### Reviewer Comments

In the simulations how do the authors implement a genotype-phenotype map?

##### Author's response

In our theory, one does not need to impose a specific mapping. Instead, the rate equations governing gene expression dynamics provide a relation from a given gene regulatory (or catalytic reaction) network to expression levels of genes (or protein concentrations). The genotype corresponds to each network structure, while the expression levels give a phenotype. Gene expression dynamics governed by the genotype, i.e., the network, lead to the phenotype, and thus genotype-phenotype is determined as a result of development (gene expression) dynamics. However, in our recent study using GA as mentioned above, we considered the mutation of the genotype (network) to choose a higher fitness value given by the phenotype, to increase heterogeneity of expression patterns over cells.

#### Reviewer Comments

Why does the stem cell not resemble many different differentiated cells as is suggested by figure 1?

##### Author's response

Experimental observations show that the state of a stem cell "resembles" the states of differentiated cells to some degree. In a stem cell, large numbers of genes, including lineage-specific genes of several differentiated types, are weakly expressed, while differentiated cells express a smaller number of lineage-specific genes strongly. Both our simulation results and experimental observations suggest that the state of stem cells occupies an intermediate region between differentiated cells in the phase space as presented in Figure 1.

#### Reviewer Comments

Also, with regard to figure 1, I do not think it currently makes sense. The dynamics are changing but the epigenetic landscapes are invariant. The authors seem to suggest in the figure that the only difference between states/types is the instantaneous position of the expression vector. I think this needs to be redrawn to be consistent with the hypothesis.

##### Author's response

The representation of epigenetic landscape for cellular states is simplified by discarding temporal variation at each attractor. The landscape represents only attraction to each attractor, while the temporal change of cellular state at each attractor is not represented therein. If needed one can add another dimension (or several dimensions) orthogonal to the axis of the landscape, to represent (chaotic) dynamics of each attracted cellular state. Here that dimension is discarded, following the representation by Waddington.

### Reviewer's report 2

Dr. Jeroen van Zon (Department of Mathematics, Imperial College London, London, UK)

#### Reviewer Comments

In their 'Hypothesis' the authors discuss a model explaining how pluripotent stem cells differentiate into less-potent, committed cells. In particular, they set out to explain the following set of recent experimental observations: 1) critical stem cell markers show a remarkable stochastic variation in a clonal population of uncommitted stem cells, 2) a subpopulation of these cells with a narrow (e.g. high) level of marker will over time return to the original, wide distribution of marker level, 3) differentiated cells express a much narrower range of genes and exhibit less variation.

The authors propose that the underlying networks exhibit chaotic, oscillating dynamics, leading to the observed, slow variation in protein levels. Differentiation occurs by cell-to-cell interaction, forcing the cell dynamics from the orginal attractor, representing the undifferentiated state, into one of several different attractors, corresponding to differentiated states. The subject is highly relevant and timely, but unfortunately I am skeptical of the model the authors provide. In particular, I am not convinced of the central role of chaotic oscillators in their hypothesis, as I will clarify below.

My main issue is the central role for chaotic dynamics in differentiation. The authors use chaotic dynamics of the underlying transcriptional network to explain the observed stochastic variation in levels of stem cell markers. However, chaotic transcriptional dynamics seems exceedingly rare in nature. I am not aware of any transcriptional system (either natural or synthetic) that has been shown convincingly to exhibit chaotic dynamics. Instead, the vast majority of transcriptional networks seem constructed from 'simple' multi-stable switches. Transcriptional networks that function as non-linear oscillators are also known, but none of them (as far as I know) exhibit chaotic dynamics. The type of system that the authors simulate, where a large number of randomly connected genes exhibit chaotic oscillations, are not at all well supported by experimental observations.

##### Author's response

When we proposed the original hypothesis about ten years ago [2,3], high-resolution transcriptional measurements at the single-cell level were almost impossible, and thus the oscillation and heterogeneity in the gene expression dynamics we proposed did not attract much attention, as they were not observed at that time. However, at present, many studies have revealed that oscillatory behavior and heterogeneity are widely seen in the expression dynamics of stem cells (*e.g*., [19]). Although chaotic oscillation in such expression dynamics is not supported experimentally so far, we expect that further advances in accuracy and temporal resolution in the expression measurement will provide evidence of chaotic dynamics. For example, recent stem cell studies have shown heterogeneity in the expression levels of some genes [5-7]. However, the origin of such heterogeneity still remains unclear. By controlling the culture condition to allow for longer stable measurement of gene expression with higher resolution, one can confirm if such heterogeneity originates in chaotic dynamics, rather than in simple noise.

#### Reviewer Comments

In addition, I see no reason why 'simple' multi-stable switches cannot explain the experimental observations described above. The results in Chang et al., for instance, seem very similar to results obtained by the Van Oudenaarden group in yeast cells (Acar et al. Nature 435, 228 (2005)). Here, the authors have constucted a simple bistable switch using the yeast galactose network. Stochastic fluctuations allow cells to randomly switch from one stable state to another. This leads to very similar behaviour as in Chang et al.: a subpopulation of cells with high or low concentrations of a reporter gene will recover the full, original population over time (compare Fig. 2 of Chang et al. with Fig. 4 of Acar et al.) One can imagine how in such a system an external signal acting on the feedback loops can stabilize one stable state over another, effectively committing the cell to a particular 'fate'. Such an explanation of cell differentiation seems much more in line with current knowledge of transcriptional regulation and stochastic gene expression than the highly speculative hypothesis of large-scale chaotic gene expression.

I think the authors should at the very least motivate more persuasively why the more exotic hypothesis of chaotic gene expression is preferred over the established picture of multistable switches. The one point in the 'hypothesis' where this is currently discussed, the first paragraph of 'Implications of the hypothesis', is unclear. I am not sure what the authors mean by 'rate of differentiation' and how chaotic gene expression regulates that better than stochastic fluctuations. From the rest of the paragraph, it suggests the authors refer to the ability of stem cells to recover the full distribution of gene expression over time from a particular subpopulation. However, as I discussed above, this can be explained naturally by stochastic fluctuations in a multistable system.

##### Author's response

As the reviewer pointed out, a simple multistable system with noise can explain a switch between states and recovery to the original distribution by random switching. For example, it can probably explain differentiation in bacteria [22]. However, application of this mechanism to a stem cell has several problems, with regard to the robustness of the number distribution of each cell type, the need for fine-tuning of the noise amplitude, and further difficulty in achieving more than two cell types, as already described in the reply to the third question of the first reviewer. In contrast, our mechanism of interaction-based differentiation from chaotic dynamics overcomes these difficulties naturally without the need of fine-tuning parameters. Indeed, as mentioned in the reply to the first reviewer and also described in the revised text, most networks evolved to achieve multiple cell types adopt chaotic dynamics, rather than a metastable system.

#### Reviewer Comments

In addition, the authors speculate that differentiation of stem cells occurs mainly through cell-cell interactions by a kind of synchronization phenomenon. However, in many cases stem cells differentiate in response to external signals, for instance by moving out of a stem cell niche (e.g. germ stem cells in C. elegans or stem cells in mammalian intestinal crypts). In other cases, differentiation occurs by asymmetric division of stem cells into a stem cell and a differentiated cell. I am not sure whether the model presented by the authors still shows proper differentiation in such different scenarios.

##### Author's response

We agree that external signals play important roles to control differentiation in the developmental process. However, if we assume that all differentiation processes are controlled by external signals, an essential question arises: how are such external signals generated? Indeed, there are mechanisms to provide such signals by surrounding cells. They are generated from inside the system through cell-cell interactions. In fact, there are several established experimental results supporting the idea that signals controlling differentiation are generated within the system. For example, mammalian embryonic stem cells can differentiate into all three germ layers when they form a spherical structure called the embryoid body (EB), in which differentiated tissues generally appear without any signals given from the outside of the body. Another classic example is a callus of undifferentiated plant cells which forms a complete body without any external signals for positional information. In such systems, signals to control differentiations are not provided from the outside, but emerge as a result of cell-cell interactions. Our hypothesis proposed here includes such emergence of signals through cell-cell interactions. In our previous paper we discussed the emergence of positional information, by using simulations of the same multicellular dynamic model with spatial interactions [23]. In that study, we found that through cell-cell interaction, undifferentiated stem cells differentiate, and a spatial interaction between cells with multiple cell types generates a gradient of signals in the environment. Then, this gradient of signals controls the further developmental process. Here, the positional information from the gradient of signals spontaneously emerges without any external control. Our results suggest that such a gradient of signals for differentiation is generated and maintained through cell-cell interactions within the system. Also, an important point here is that the signals maintained by the cell-cell interaction are regulated spontaneously to achieve robustness of the cell population, as described in our papers.

As for asymmetric division, we note that the differentiation process of our dynamic mechanism is quite fast, in contrast to a multistability mechanism. Hence right after the division, the state differentiation has already taken place, so that the division could be regarded as asymmetric.

### Reviewer's report 3

Dr. William S. Hlavacek (Theoretical Division and Center for Nonlinear Studies, Los Alamos National Laboratory, Los Alamos, NM, USA)

#### Reviewer Comments

The authors argue that the genetic regulatory network of a pluripotent stem cell is marked by chaotic dynamics, whereas the network of a differentiated cell is marked by less complex, less rich dynamics. It seems that the benefits of this situation would be twofold: a larger region of phase space would be accessible from a strange attractor than a fixed point, say, and as a result, a stem cell would presumably be able to differentiate into a greater variety of cell types, and differentiation would be more irreversible, i.e., more committed and robust once the process of differentiation is initiated. However, the authors suggest that the advantage is regulation of the rate of differentiation. I do not understand how the authors reach this conclusion, even after reading Ref. 3. I also do not understand what the authors are trying to say about the role of cell-cell interactions. Incidentally, the hypothesis put forward in this manuscript seems to be similar to ideas that the authors have considered in earlier work. What is new here?

##### Author's response

The novelty in the present paper is stressed in the first answer for the first reviewer. To sum up again: i) we give novel interpretations of recent experimental results, ii) we propose possible ways to validate our hypothesis by using experimental techniques developed recently, and iii) we provide some of our recent simulation results, in which more biologically plausible assumptions are used. They support ubiquity of our differentiation mechanism. Also we have added a remark on our recent simulations demonstrating that most networks evolved to have multiple cell types indeed show chaotic gene expression at the initial stage of development, which plays the role of a stem cell. We believe that these topics provide a novel insight into stem cell dynamics.

#### Reviewer Comments

The authors provide guidance on how to test their hypothesis, emphasizing the need to measure single-cell gene expression dynamics, and they discuss the implications of their hypothesis. For example, the authors indicate that their hypothesis suggests that recovery of pluripotency can be achieved by activating the expression of multiple genes. It seems that this prediction would be difficult to test. How many genes should be activated? Which genes should be activated? Is it possible to activate a large number of genes and fail to recover pluripotency? With respect to the issue of testing for chaotic dynamics given single-cell data, I wonder how feasible this exercise might be in practice. It can be difficult to distinguish a stochastic process from a chaotic one, especially when noise is considered. Could the authors generate simulated data sets using models that produce chaotic and non-chaotic dynamics and walk through the steps that would be necessary to correctly identify the two types of dynamics in the face of noise?

##### Author's response

As you pointed out, it is rather difficult at the moment to predict which genes should be activated to recover pluripotency. To predict genes whose activation lead to recovery of pluripotency, further advances in both theoretical and experimental studies are necessary. Note, however, that pluripotency is tightly correlated with chaotic oscillation of some gene expression level according to our theory. Hence, by measuring time-series of expression levels of many genes involved in a stem cell's differentiation process, and detecting which genes exhibit (chaotic) oscillation, we may expect that such genes are, at least, candidates to recover pluripotency. Further simulation studies in models with appropriate gene regulation are also important to relate them to the experimentally observed dynamic gene expression.

For the experimental detection of chaos in the expression dynamics, we expect that standard techniques in time-series analysis will be helpful. There are several previous studies in which the existence of chaotic dynamics was verified by analyzing experimentally obtained time-series [24]. Even from noisy data sets with a relatively small size, appropriate statistical evaluation on time series can distinguish chaotic dynamics from experimental or intrinsic noise. When sufficient data on time-series of gene expression levels of individual cells in a well controlled culture condition are available, even if some noise exists, we expect that evidence of chaotic dynamics in the expression levels will be uncovered in future.

#### Reviewer Comments

The idea that dynamical system behavior is important in differentiation and that chaotic dynamics may be involved reminds me of theoretical work on neural networks, in particular work of Hopfield and Sompolinsky in the 80's and 90's. I wonder if the authors also see a connection. If so, could this earlier work be useful for testing the authors' hypothesis and investigating its implications? Finally, I am somewhat skeptical that differentiation (or regulation of cellular activities in general) could depend on chaos, although the thought is intriguing. Could the authors speculate on the possible molecular mechanisms responsible for chaos and the role of chaos in differentiation and how these mechanisms might have arisen through evolution to have the function suggested by the authors' hypothesis? I would be less skeptical if the authors could give examples of cellular regulatory systems that have been confirmed experimentally to exhibit chaotic dynamics. Are there cases where chaos has been shown not just theoretically but experimentally to play an important role in cellular regulation?

##### Author's response

Recently, we have performed extensive simulations of an interacting cell model, in which each cell has gene regulatory dynamics represented as on/off switch dynamics as in neural network models. In the simulations, the gene regulatory networks which can generate multiple cell types are selected by using a genetic algorithm (GA) with cell type heterogeneity as the fitness function. As also mentioned in the reply to the third question of the first reviewer, we have found that the selected regulatory networks ubiquitously show chaotic dynamics in their stem-type cells, from which cell differentiation progresses following our theory. This result suggests that when cell type differentiation is favorable for organism survival, chaotic expression dynamics are likely to be selected through evolution. Thus, even though chaotic dynamics in gene expression levels are not confirmed in experiments at the moment, we expect that further investigation of time-series analysis of gene expression levels at single cell level will reveal the existence of chaotic expression dynamics. In the revised manuscript, we have added some brief remarks on the recent results in the last paragraph of the "Testing the hypothesis" section.
